# Electronic Implementation of Patient-Reported Outcome Measures in Primary Health Care: Mixed Methods Systematic Review

**DOI:** 10.2196/63639

**Published:** 2025-05-05

**Authors:** Maxime Sasseville, Wilfried Supper, Jean-Baptiste Gartner, Géraldine Layani, Samira Amil, Peter Sheffield, Marie-Pierre Gagnon, Catherine Hudon, Sylvie Lambert, Eugène Attisso, Steven Ouellet, Mylaine Breton, Marie-Eve Poitras, Pierre-Henri Roux-Lévy, James Plaisimond, Frédéric Bergeron, Rachelle Ashcroft, Sabrina T. Wong, Antoine Groulx, Jean-Sébastien Paquette, Natasha D'Anjou, Sylviane Langlois, Annie LeBlanc

**Affiliations:** 1 Faculty of Medicine Université Laval Quebec City, QC Canada; 2 Faculty of Medicine Université de Montréal Montréal, QC Canada; 3 Factor-Inwentash Faculty of Social Work University of Toronto Toronto, QC Canada; 4 Faculty of Medicine and Health Sciences Université de Sherbrooke Sherbrooke, QC Canada; 5 Faculty of Nursing Université McGill Montréal, QC Canada; 6 Centre de recherche de St. Mary's Montréal, QC Canada; 7 UBC Centre for Health Services and Policy Research and School of Nursing Vancouver, BC Canada; 8 Patient Partner Université Laval Québec, QC Canada

**Keywords:** patient-reported outcomes measures, digital health, implementation science, systematic review, PRISMA

## Abstract

**Background:**

Managing chronic diseases remains a critical challenge in primary health care (PHC) across the Organization for Economic Co-operation and Development countries. Electronic patient-reported outcome measures (ePROMs) are emerging as valuable tools for enhancing patient engagement, facilitating clinical decision-making, and improving health outcomes. However, their implementation in PHC remains limited, with significant variability in effectiveness and adoption.

**Objective:**

This systematic review aimed to assess the implementation and effectiveness of ePROMs in chronic disease management within PHC settings and to identify key barriers and facilitators influencing their integration.

**Methods:**

A mixed methods systematic review was conducted following the Cochrane Methods and PRISMA (Preferred Reporting Items for Systematic Reviews and Meta-Analyses) guidelines. We included studies that implemented ePROMs among adults for chronic disease management in PHC. The extracted data included patient health outcomes, provider workflow implications, implementation factors, and cost considerations. The reach, effectiveness, adoption, implementation, and maintenance framework was used.

**Results:**

Our search yielded 12,525 references, from which 22 (0.18%) studies were included after screening and exclusions. These studies, primarily conducted in the United States (n=9, 41%) and Canada (n=8, 36%), covered various chronic diseases and used diverse ePROM tools, predominantly mobile apps (n=9, 41%). While some studies (n=10, 45%) reported improvements in patient health outcomes and self-management, others (n=12, 55%) indicated no significant change. Key barriers included digital literacy gaps, integration challenges within clinical workflows, and increased provider workload. Facilitators included strong patient-provider relationships, personalized interventions, and technical support for users. While some studies (n=10, 45%) demonstrated improved patient engagement and self-management, long-term cost-effectiveness and sustainability remain uncertain.

**Conclusions:**

Success in implementing ePROMs in PHC appears to hinge on addressing digital literacy, ensuring personalization and meaningful patient-provider interactions, carefully integrating technology into clinical workflows, and conducting thorough research on their long-term impacts and cost-effectiveness. Future efforts should focus on these areas to fully realize the benefits of digital health technologies for patients, providers, and health care systems.

**Trial Registration:**

PROSPERO CRD42022333513; https://www.crd.york.ac.uk/PROSPERO/view/CRD42022333513

**International Registered Report Identifier (IRRID):**

RR2-10.2196/48155

## Introduction

The World Health Organization advocates for universal access to primary health care (PHC), a critical first point of contact within health care systems in the Organization for Economic Co-operation and Development countries, despite ongoing access limitations [1-4].

Chronic diseases, primarily managed in this setting, continue to be leading causes of mortality and morbidity in these countries [5,6]. The Canadian Institute of Health Research promotes the use of patient-reported outcome measures (PROMs) to enhance patient experience, clinical outcomes, and health care efficiency [7]. While PROMs have been widely implemented in hospital settings, their use in primary care remains underexplored. PROMs are assessments of a patient’s health status based on their own perceptions, without input from a third party. These reports are collected using validated questionnaires that quantify aspects such as quality of life, disease management, daily functioning, and symptoms [8]. For more than a decade, governments in several countries have funded initiatives to develop, implement, and use PROMs in hospitals [9] and PHC settings [10]. Optimal implementation and use of PROMs are associated with clinical benefits, such as improved communication with patients, better adaptation of health care to patient needs, and shorter consultation times [9].

The emergence of digital tools has a great potential for the implementation and use of PROMs in health care settings [11]. Digital methods, compared to traditional pen-and-paper approaches, enhance data collection quality, reduce costs, support clinical decision-making, and are better received by patients [12]. Several systematic reviews have identified the barriers and facilitators associated with their implementation in health care systems and evaluated the effectiveness of digitally collected electronic PROMs (ePROMs) [8,13,14]. In oncology, the main barriers to implementation are increased workload and inadequate technological infrastructure [8,14]. In terms of effects, these systematic reviews note that there is a fairly wide divergence between the results of the studies that have been identified. However, in oncology and pediatrics, the implementation of ePROMs is associated with an increase in quality of life and patient satisfaction [8,13]. While systematic reviews have identified impacts, barriers, and facilitators for ePROMs implementation in specialized settings, these findings have limited transferability to PHC due to differences in patient populations and clinical workflows; for example, the wider age range in primary care makes adapting ePROMs to varying levels of digital literacy more challenging. The implementation of ePROMs in PHC has however the potential to provide unique benefits, particularly by supporting self-management of chronic disease symptoms [15].

This review aims to evaluate the implementation and effectiveness of ePROMs for chronic disease management in PHC, identifying associated barriers and facilitators.

## Methods

### Ethical Considerations

We conducted a systematic review of the literature according to the Cochrane Methods Group and in compliance with the PRISMA (Preferred Reporting Items for Systematic Reviews and Meta-Analyses) guidelines for its reporting (Multimedia Appendix 1) [16,17]. We registered the study protocol with the PROSPERO Systematic Review Registry (ID: CRD42022333513) [18] and have also published it [19].

### Synthesis Questions

The synthesis questions were (1) What are the effective strategies to implement ePROMs in PHC? (2) What are the challenges and barriers and facilitators to successful implementation of ePROMs in PHC? and (3) What are the outcomes of ePROMs in PHC chronic disease management?

### Eligibility Criteria

We included all types of evidence matching the following PICOS (Population, Intervention, Comparison, Outcomes, and Setting) [16]:

- Population: All studies including the implementation of an ePROM among adults for chronic disease management
- Interventions or phenomena of interest: No restrictions. We included all types of implementations, theoretical models, structures, or PROMs.
- Comparator: No restrictions
- Outcomes: We considered all outcomes reported in the studies. We sought outcomes related to patients, caregivers, health care providers, policy makers, barriers, facilitators, acceptability, feasibility, adoption, fidelity, morbidity, mortality, quality of life, satisfaction, cost, and cost-effectiveness.
- Setting: We included studies conducted exclusively in PHC settings, regardless of location, and extracted information on implementation.

We included all types of empirical studies (qualitative, quantitative, and mixed methods) published in French, English, or Spanish.

### Search Strategy

Using an iterative process, the search strategy was developed in collaboration with an experienced information specialist (FB). On August 15, 2022, we searched the following databases: MEDLINE (OVID), Embase (via Embase), CINAHL (EBSCO), and Web of Science. Considering the large number of results, we decided not to consult gray literature sources (eg, GreyNet, Grey Matters, Google, websites, and ProQuest Dissertations & Theses), as suggested in our protocol. We applied no restrictions to the search strategy, including time limit, as mentioned in our protocol due to the lack of effect in reducing the number of results. The full search strategy is available in Multimedia Appendix 2.

### Data Collection and Screening

**Figure 1:**
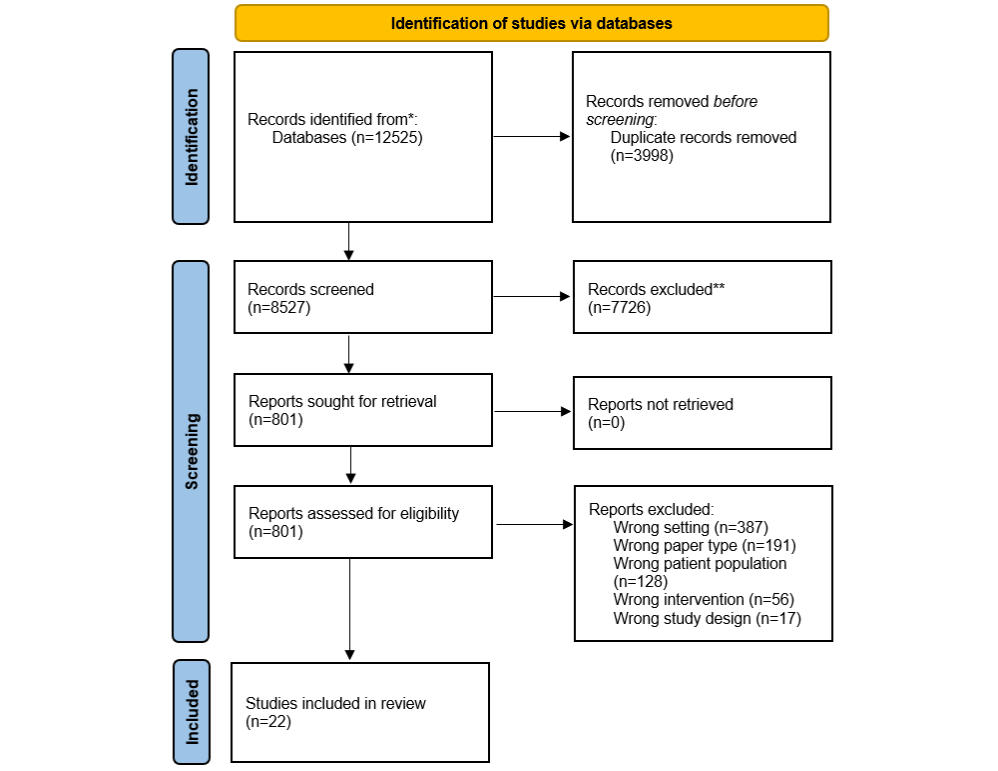
PRISMA (Preferred Reporting Items for Systematic Reviews and Meta-Analyses) flow diagram.

We exported all citations in the web-based collaboration tool Covidence, where duplicates were removed with the automated function [20]. Pairs of reviewers screened the titles and abstracts independently. We retained ambiguous or incomplete abstracts to be reviewed in full. We searched and obtained all the full texts of the selected references and imported the PDF files in Covidence. Pairs of reviewers then independently applied the inclusion criteria using the full texts following a pilot testing using the process outlined above. At any moment in the screening process, the first author helped resolve any discrepancy. All the reasons for exclusions were recorded in Covidence, and a PRISMA flowchart describes study identification, screening, inclusions, and exclusions (Figure 1) [21].

### Data Extraction and Appraisal

We extracted descriptive data (title, year of publication, authors, funding, conflicts of interests, and country), study types (published or gray literature), methodological data (design, sample size, measure constructs, and name of the instrument), setting data (clinical setting, type health professionals, and patient population), characteristics of the ePROM tools (functionality, interface and delivery platform, and integration approach), implementation data (description of implementation strategies, facilitators, and barriers), outcomes (patient health, providers workflow, and cost), and outcomes type (qualitative and quantitative). The quality of the included studies was evaluated using the Mixed Methods Appraisal Tool (MMAT), which is designed for systematic reviews that synthesize data from qualitative, quantitative, and mixed methods studies [22].

### Data Synthesis

We used the reach, effectiveness, adoption, implementation, and maintenance (RE-AIM) framework as a data analysis framework. RE-AIM has been developed to evaluate the public health impacts of interventions and has been used in systematic reviews to help structure the assessment of the different implementation factors at play in complex contexts and settings [23,24]. This framework includes 5 dimensions: reach (how willing the targeted population is to participate in the intervention; ie, ePROMs), efficacy (what is the impact of the intervention on outcomes), adoption (can this be adopted by new groups with ease and minimal changes), implementation (what are the special issues and barriers), and maintenance (can the intervention be maintained and the impact continued). The use of RE-AIM enabled us to give an overview of the parameters strengthening (review questions 1 and 2) the efficiency (review question 3) of ePROMs’ integration in PHC and its impact on outcomes.

We used a 2-phase sequential mixed methods synthesis design, that is, conduct a qualitative synthesis and use its results to inform the quantitative synthesis [25]. For phase 1 (qualitative), we summarized and described methods and approaches designed to implement and integrate ePROMs in PHC, using a thematic synthesis procedure [26]. The qualitative data synthesis produced narrative summaries of main themes, which were then classified according to the RE-AIM framework. We summarized study characteristics and methodological differences and similarities to highlight the following points: strengths and weaknesses of each implementation method, main outcomes of implementation, main resources used and their impacts, and if any trade-offs are described and their effect on the results of the study.

## Results

### Overview

The PRISMA diagram (Figure 1) shows the results of the search strategy and study selection process. Our search strategy identified 12,525 references. The exclusion of duplicates (n=3998) and the first selection stage (titles and abstracts) led us to retain 761 references. A further 749 references were excluded when the texts were read in full. In all, 22 studies met the selection criteria and were therefore selected.

### Study Characteristics

Table 1 summarizes descriptive data from the 22 selected studies; of these, 5 (23%) are mixed-design studies, 6 (27%) are qualitative studies, and 11 (50%) are quantitative studies. These 22 studies were published between 2015 and 2022, and the majority were conducted in the United States (n=9, 41%) and Canada (n=8, 36%). The number of participants included in these studies ranged between 2334 and 8, and only 3 studies had >300 participants. The aims of the studies selected align around improving clinical outcomes for patients with chronic diseases and enhancing health care delivery through better integration of patient-reported data into clinical practice. In most studies, the average age of the population was >50 years. Gender representation varied from study to study, but in the majority (16/22, 73%), women represented >50% of participants. Additional information about the intervention are presented in Multimedia Appendix 3.

The main chronic diseases population targeted are asthma (4/22, 18%), cardiovascular diseases (3/22, 14%), multimorbidity (5/22, 23%), mental health (3/22, 14%), diabetes (2/22, 9%), or complex cases of chronic disease (2/22, 9%). Regarding ePROMs, the digital tool most frequently implemented was a mobile app (9/22, 41%). The constructs of ePROMs varied between 3 main categories: general health (eg, quality of life and health status), symptom monitoring (eg, for sleep, pain, anxiety, and depression), and self-management (eg, self-efficacy and patient activation).

**Table 1:** Studies description.

Study	Country	Aim of the study	Age (y), mean	Women (%)	Targeted Constructs	Chronic diseases	Sample size, n	Study design^a^
Staeheli et al [27]	United States	Compare screening results to data derived from chart reviews of patients seen before the deployment of the screening intervention to determine the following: The rates of unrecognized and undiagnosed depression, PTSD^b^, and risky drinking (referred to collectively as “behavioral health problems” for the purposes of this study) in this patient population and Whether increased recognition of behavioral health problems in the encounter was associated with appropriate treatment and follow-up of identified patients.	Not specified but around 50	66.54	Depression, PTSD, and problem drinking	PTSD, depression, and risky drinking	275	Quantitative
Ainsworth et al [15]	United Kingdom	Assess the feasibility of a trial to evaluate a digital intervention in primary care to improve quality of life and other clinical outcomes of people with asthma, in comparison to usual care	56.6	53	Quality of life and self-monitoring	Physician-diagnosed asthma	88	Quantitative
Harle et al [28]	United States	Assess whether integrating PROs^c^ data in an EHR^d^ affects providers and patient satisfaction with chronic noncancer pain care	43.8	62	Pain interference, pain behavior, fatigue, and anger	Chronic musculoskeletal	680	Quantitative
Kroenke et al [29]	United States	Assess the effectiveness of providing patient-reported outcomes measurement information system symptom scores to clinicians on symptom outcomes	49	72	SPADE^e^	SPADE symptom	256	Quantitative
Lear et al [30]	Canada	To compare the effect of an internet-based self-management and symptom monitoring program targeted to patients with ≥2 chronic diseases (internet chronic disease management) with usual care on hospitalizations over a 2-y period	70.5	38.40	Self-monitoring and self-management	≥2 chronic conditions	229	Quantitative
Miranda et al [31]	Canada	Estimate the cost of an ePRO^f^ tool and examine whether the benefits gained from the tool outweighed its costs from the perspective of Canada’s publicly funded health care system	Not specified but they recruiter patients ≥60 years old	Not specified	Global health	≥2 chronic conditions	45	Quantitative
Baron and Duffecy [32]	United States	Evaluate the feasibility and preliminary efficacy of a technology-assisted sleep extension intervention among individuals with prehypertension or stage 1 hypertension on sleep, blood pressure, and PROs.	45.8	50	Sleep quality	24 h ambulatory blood pressure	16	Quantitative
Owen-Smith et al [33]	United States	Describe the infrastructure designed to automate PRO data collection Compare study-enhanced PRO completion rates to those in clinical care Assess patient response rates based on the PRO administration method and their sociodemographic or clinical characteristics.	Not specified	Not specified	Pain	Chronic pain	632	Quantitative
Ramallo-Fariña et al [34]	Spain	Assess the effectiveness of different interventions of knowledge transfer and behavior modification to improve the PROMs^g^ of patients with T2DM^h^ in the long term	55.7	51.90	Diabetes empowerment	Type 2 diabetes mellitus at least 1 y before	2334	Quantitative
Tamisier et al [35]	France	Assess the effect of a multimodal telemonitoring intervention on treatment adherence, quality of life, and functional status in symptomatic patients with OSA^i^ and low cardiovascular risk	50.6	63.59	Quality of life	OSA	206	Quantitative
Trick et al [36]	United States	Evaluate time burden for patients and factors associated with response times for an audio computer-assisted self-interview system integrated into the clinical workflow	57.0	58	General health	Chronic illness	1670	Quantitative
Yanicelli et al [37]	Argentina	Validate a home telemonitoring system for the first time in a real setting; its effectiveness to improve self-care and treatment adherence of patients with HF^j^ was assessed through a randomized controlled clinical trial in Argentina	52	20	Weight, blood pressure, heart rate, and symptoms	HF	30	Quantitative
Bezerra Giordan et al [38]	Australia	Explore the patient’s and primary care clinician’s perspective on the facilitators and barriers to using mobile apps, as well as desired features, to support HF self-management	69	66.66	Self-management	Confirmed diagnosis of HF	12	Qualitative
Steele Gray et al [39]	Canada	Tested the usability and feasibility of adopting the ePRO tool into a single interdisciplinary primary health care practice. The Fit between Individuals, Fask, and Technology framework was used to guide our assessment and explore whether the ePRO tool is feasible for adoption in interdisciplinary primary health care practices and usable from both the patient and provider perspectives	58	54.54	General Health Scale and pain	≥2 chronic conditions	14	Qualitative
Hans et al [40]	Canada	How the ePRO mobile app and portal system, designed to capture patient-reported measures to support self-management, affected primary care provider workflows	56.3	50	Global health and pain interference	≥2 chronic conditions	18	Qualitative
Harle et al [28]	United States	Identify the lessons learned in overcoming barriers to collecting and integrating PROs in an EHR	Not specified	Not specified	Pain interference, pain behavior, fatigue, and physical function	Chronic pain	12	Qualitative
Irfan Khan et al [41]	Canada	Explore the experience and expectations of patients with multimorbidity and their providers around the use of the ePRO tool in supporting self-management efforts	58	50	Global health, pain interference, and generalized anxiety	Multimorbid	9	Qualitative
Schoenthaler et al [42]	United States	Apply a systematic, user-centered design approach to develop i-Matter (investigating a mobile health texting tool for embedding patient-reported data into diabetes management), a theory-driven, mobile PRO system for patients with T2D and their primary care providers.	62.5	67	Diabetes self-management	T2D for ≥6 mo	12	Qualitative
Steele Gray et al [43]	Canada	What are the contexts, processes, and outcomes most relevant to the ePRO intervention? What are the central (critical to achieving outcomes) versus peripheral (less critical and potentially context dependent) mechanisms that underpin the content theory of the ePRO intervention?	Not clear but around 63	68.75	Goal attainment	Not specified	16	Mixed methods
Ahmed et al, 2021 [44]	Canada	Identify the potential barriers and enablers to using PROMs in primary care LBP^k^ clinical practice from the perspective of health care team members Develop a theory-based tailored knowledge translation intervention to facilitate the use of PROMs in interdisciplinary clinical practice	39	39	Not specified	LBP	18	Mixed methods
Steele Gray et al [45]	Canada	Evaluate the implementation and effectiveness of the ePRO mobile app and portal system designed to enable goal-oriented care delivery in interprofessional primary care practices	68.7	65.22	Goal setting, self-management, mental health, and social health	Older adults with complex needs	44	Mixed methods
Bauer et al [46]	United States	Assess the feasibility and acceptability of a mobile health platform supporting collaborative care	Not specified but around 35	59	Depressive and anxiety symptoms	Depression or anxiety disorder	17	Mixed methods

^a^Qualitative: studies primarily collecting and analyzing nonnumerical data (eg, interviews and focus groups), quantitative: studies primarily collecting and analyzing numerical data (eg, randomized controlled trials and cohort and cross‐sectional studies), and mixed methods: studies integrating both quantitative and qualitative approaches. We also reference these criteria in the Data Extraction and Appraisal section for clarity.

^b^PTSD: posttraumatic stress disorder.

^c^PRO: patient-reported outcome.

^d^EHR: electronic health record.

^e^SPADE: sleep problems, pain, anxiety, depression, and low energy or fatigue.

^f^ePRO: electronic patient-reported outcome.

^g^PROM: patient-reported outcome measure.

^h^T2DM: type 2 diabetes mellitus.

^i^OSA: obstructive sleep apnea.

^j^HF: heart failure.

^k^LBP: lower back pain.

### Assessment of Studies’ Methodological Quality

The evaluation of the studies’ methodological quality shows that the majority (15/22, 68%) received a score of ≥80%. Specifically, 38% (8/22) of the studies scored 100%, 33% (7/22) of the studies scored between 80% and 95%, and 19% (4/22) of the studies scored between 60% and 75%. Only 2 studies scored ≤40% (Table 2). While most of the included studies (15/22, 68%) met a high standard of methodological rigor (≥80% on the MMAT), this does not entirely eliminate concerns about heterogeneity, smaller sample sizes, and diverse outcome measures. Higher-quality studies generally provided robust justifications for their choice of ePROM tools and specified clear implementation processes (eg, training sessions and technical support). In contrast, studies with lower MMAT scores often lacked detail on how they integrated ePROMs into existing workflows or offered minimal information on training protocols. These distinctions may influence both the internal validity and generalizability of the findings.

**Table 2:** Assessment of studies’ methodological quality using the Mixed Methods Appraisal Tool (MMAT).

Study	MMAT items																																		
	1.1	1.2	1.3	1.4	1.5		2.1		2.2		2.3		2.4		2.5		3.1		3.2		3.3		3.4		3.5		5.1		5.2		5.3		5.4		5.5
Staeheli et al [27]	—^a^	—	—	—	—	—		—		—		—		—		1^b^		1		1		0^c^		1		—		—		—		—		—	
Bauer et al [46]	1	1	1	1	1	—		—		—		—		—		0		1		0		0		0		1		1		1		1		1	
Trick et al [36]	—	—	—	—	—	—		—		—		—		—		1		1		0		1		1		—		—		—		—		—	
Owen-Smith et al [33]	—	—	—	—	—	—		—		—		—		—		0		1		1		0		1		—		—		—		—		—	
Steele Gray et al [43]	1	1	1	1	1	1		0		1		0		1		—		—		—		—		—		1		1		1		0		0	
Bezerra Giordan et al [38]	1	1	1	1	1	—		—		—		—		—		—		—		—		—		—		—		—		—		—		—	
Schoenthaler et al [42]	1	1	1	1	1	—		—		—		—		—		—		—		—		—		—		—		—		—		—		—	
Ahmed et al [44]	1	1	0	1	1	—		—		—		—		—		—		—		—		—		—		—		—		—		—		—	
Kroenke et al [29]	—	—	—	—	—	1		1		1		0		1		—		—		—		—		—		—		—		—		—		—	
Lear et al [30]	—	—	—	—	—	1		1		1		1		1		—		—		—		—		—		—		—		—		—		—	
Baron et al [32]	—	—	—	—	—	0		0		0		0		1		—		—		—		—		—		—		—		—		—		—	
Ramallo-Fariña et al [34]	—	—	—	—	—	1		1		1		0		1		—		—		—		—		—		—		—		—		—		—	
Tamisier et al [35]	—	—	—	—	—	1		0		1		1		1		—		—		—		—		—		—		—		—		—		—	
Yanicelli et al [37]	—	—	—	—	—	1		0		0		0		1		—		—		—		—		—		—		—		—		—		—	
Ainsworth et al [15], 2019	—	—	—	—	—	1		1		1		1		1		—		—		—		—		—		—		—		—		—		—	
Steele Gray et al [45]	1	1	1	1	1	1		1		1		1		1		—		—		—		—		—		1		1		1		0		1	
Steele Gray et al [39]	1	1	1	1	1	—		—		—		—		—		—		—		—		—		—		—		—		—		—		—	
Hans et al [40]	1	1	1	1	1	—		—		—		—		—		—		—		—		—		—		—		—		—		—		—	
Harle et al [28]	1	1	1	1	1	—		—		—		—		—		—		—		—		—		—		—		—		—		—		—	
Harle et al [28]	—	—	—	—	—	1		0		1		0		1		—		—		—		—		—		—		—		—		—		—	
Irfan Khan et al [41]	1	1	1	1	1	—		—		—		—		—		—		—		—		—		—		—		—		—		—		—	

^a^Not applicable.

^b^This criterion was met.

^c^This criterion was not met.

### Qualitative Results

#### Overview

We present the entire thematic organization and representatives quotes of the qualitative studies structured according to the RE-AIM model in Table 3.

**Table 3:** Qualitative themes of the barriers and facilitators according to the reach, effectiveness, adoption, implementation, and maintenance model.

Study		Reach, effectiveness, adoption, implementation, and maintenance	Actor	Categories	Themes	Representative quotes
**Barriers**						
	Steele Gray et al [39]	Reach	Patient	Communication	Lack of patient-provider interaction	“However, some patients reported feeling isolated with the mobile device, and felt that the tool could become a replacement for in person consultation.”
	Bezerra Giordan et al [38]	Reach	Patients, clinicians, and organizations	Digital literacy	Lack of digital literacy of patients and providers’ lack of training	“Patients mentioned seeing themselves as not being tech-savvy enough to use an app [mostly due to a perceived age barrier] and expected it would take them a long time and effort to learn how to use it properly.”
	Irfan Khan et al [41]	Efficacy	Patient	Communication	Lack of feedback from providers	“Patients identified gaps in the tool’s ability to promote self-efficacy in terms of the adherence to self-regulation activities because of limited feedback on their progress from providers.”
	Harle et al [28]	Efficacy	Providers	Treatment	Take time away from treatment	“Discussing these issues (psychological data on depression and anxiety) could harm care quality by diverting their attention away from acute problems that they judged to be more relevant at a given visit or more aligned with their clinical expertise.”
	Steele Gray et al [39]	Efficacy	Providers	Workload	Increase workload	“Finally, some providers felt that they may be liable for monitoring patients during out-of-office hours, which would require additional time and resources.”
	Irfan Khan et al [41]	Adoption	Patients, clinicians, and organizations	Adaptation	Reticence to adopt new practices, lack of personalization, and lack of alignment with clinical reality	“In addition, patients stressed the need for greater personalization and customizability of goals and monitoring protocols. The questions in the ePRO tool appeared to lack the depth that was considered vital to incorporating patient context into self-management activities.”
	Schoenthaler et al [42]	Implementation	Patient	ePROM^a^ digital solution	Confidentiality, difficult data entry, difficult use, retrieval of information, data presentation, technical issues, and no technical support	“Several patients had difficulty reading the bar graphs of PROs that were collected biweekly (eg, quality of life) and recommended changing the items to weekly measures to be consistent with other PROs.”
	Steele Gray et al [39]	Implementation	Patients, clinicians, and organizations	Workflow	Workflow integration and data overload	“After the initial visit, provider participants reported experiencing difficulty incorporating patient data into their workflow in terms of: (1) increased charting time required to input data into the provider’s EMR and (2) being able to view data in manageable chunks.”
	Bauer et al [46]	Maintenance	Patient	Adaptation	Lack of personalization of system features and sensitivity to change of PROM^b^	“Patients’ desire for more personalized features. Patients wanted to customize the app to meet their individual needs, for example, by adjusting the timing of prompts, the types of symptoms they were reporting on, or the frequency or content of health tips.”
	Irfan Khan et al [41]	Maintenance	Patient	Relationship	Lack of patient-provider interaction	“Patient expectations around social facilitation and social support indicated that they were expecting a more active role in self-management efforts from providers. Patients felt the tool should supplement patient-provider interaction through regular feedback and encouragement as an ‘add-on’ to existing in-person appointments rather than a replacement for in-person interaction and consults with their providers.”
	Bezerra Giordan et al [38]	Maintenance	Patients, clinicians, and organizations	Burden of treatment	Increased treatment burden	“Participants mentioned the use of an app could be associated with an increase in the burden of managing heart failure. learning how to use the app and remembering to use it could be seen as an additional responsibility in people’s already busy lives, which could be demotivating and lead to a lack of interest and decreased willingness to use the app over time.”
	Hans et al [40]	Maintenance	Providers	Workload	Increase workload	“Providers questioned whether the app would actually improve workflow functions or simply add another task. Multiple providers expressed their concerns with incorporating the ePRO into their daily visit routine.”
**Facilitators**						
	Steele Gray et al [45]	Reach	Providers and patient	Relationship	Existing patient-provider relationship	“The meaningfulness of the ePRO tool was reliant to strong relationships between patients and providers (enabling collective action)”
	Irfan Khan et al [41]	Reach	All participants	Digital literacy	Digital training and digital literacy	“The technology partner (QoC Health) offered providers two 1-hour hands-on training sessions (facilitated by the research team) on the mobile phone app and portal to provide a walk-through of the ePRO tool before starting the study, whereas, patients received one-on-one training through a 30-minute hands-on session with a member of the research team at the time when patients gave consent to participate in this study.”
	Ahmed et al [44]	Reach	Providers	ePROM literacy	Score interpretation	“Participants agreed that having the knowledge and skills to interpret PROM scores is required for clinicians to be able to use PROMs in clinical care for management of LBP.”
	Irfan Khan et al [41]	Efficacy	Patient and providers	Self-management	Improved self-management, self-efficacy, symptoms management, goal setting, and treatment quality	“Patients acknowledged the potential of the ePRO tool in building capacity to support self-management in a team-based care environment by helping to better distribute the workload across providers to meet the evolving needs of patients.”
	Bezerra Giordan et al [38]	Efficacy	Patients, providers, and organizations	Communication	Interactivity, timely action, team problem-solving, goal setting, patient-provider communication, and patient-oriented treatment	“Participants mentioned that the ability for both patients and clinicians to monitor signs of deterioration allowed for timely action.”
	Schoenthaler et al [42]	Efficacy	Providers	ePROM digital solution	Data presentation	“Providers want PRO data that are specific and actionable and can help them focus the clinic visit on what is most important for their T2D patients’ care.”
	Irfan Khan et al [41]	Efficacy	Providers	Treatment	Treatment quality	“Providers emphasized the value of the ePRO tool in helping to generate insights into underlying patient context (ie, patient preferences and readiness) to offer a fulsome sense of how patients are coping, and thereby adjust goals and self-management activities as needed.”
	Hans et al [40]	Efficacy	Providers	Workload	Reduced resource use and time saved in data retrieval during encounters	“Providers reported that the app presented an additional resource that they could leverage to quickly orient themselves to their patients’ wellbeing.”
	Schoenthaler et al [42]	Adoption	Patient and providers	ePROM digital solution	Availability of the data collection, Interoperability, and data presentation for decision-making	“These included defining a threshold that patients’ data can fall above or below and depicting it in a way that makes it easily detectible and depicting it in a way that makes it easily detectible and actionable, using bar graphs to show directionality, including icons or coloring schemes in addition to PRO labels that enhance the readability of the report, and, including summary data in percentages or raw numbers to show the patient’s progress over time.”
	Bezerra Giordan et al [38]	Adoption	Patient	Introduction	Introduction after an exacerbation	“However, this barrier could be overcome by introducing the app soon after an exacerbation, when they might be more willing to improve their self-management practices.”
	Schoenthaler et al [42]	Implementation	Providers	Communication	Patient-oriented treatment	“Similar to patients, they felt that the insight messages were helpful for interpreting the data and prompting behavioral changes.”
	Schoenthaler et al [42]	Implementation	Providers and patient	ePROM digital solution	Data presentation, collection of only relevant patient data, and comprehensible user interfaces	“Patients preferred layouts that used darker fonts and lighter background colors to help make the text easier to read. All patients viewed the color-coded schema favorably because it helped draw attention to the most important aspects of the report and made the data easy to interpret.”
	Schoenthaler et al [42]	Implementation	Providers and patient	Psychometrics	Valid PROM and sensitivity to change	“PROs should show variability in patients’ responses over time and be actionable by both patients and providers.”
	Steele Gray et al [39]	Maintenance	Providers	Adaptation	ePROMs process not aligned with clinical reality	“To improve effectiveness (and efficiency), providers wanted the tool to fit better with their existing workflows and programs, for example, through better alignment with creation of SMART goals for patients or allowing for monitoring protocols that aligned with goals of existing chronic disease management programs.”
	Steele Gray et al [39]	Maintenance	Patient and providers	Communication	Peer support, feedback aligned with needs, and automated personalized milestones	“Tool-enabled feedback, particularly from peers, was also viewed to offer encouragement on progress toward goal attainment by way of a shared experience.”
	Steele Gray et al [39]	Maintenance	Providers and patient	ePROM digital solution	Longitudinal training, patient prompting, technical support, and interoperability	“The meaningfulness of the ePRO tool was reliant to consistent positive assessments of the tool’s utility (regular reflexive monitoring).”

^a^ePROM: electronic patient-reported outcome measure.

^b^PROM: patient-reported outcome measure.

#### Digital Literacy and Training

The data highlight challenges with the lack of digital literacy among patients and providers, alongside insufficient training for providers [38,43]. These issues affected various aspects of the RE-AIM framework, notably impacting reach, adoption, and implementation. Addressing digital literacy through targeted training sessions for both patients and providers emerges as a facilitator by enhancing the usability and acceptance of ePROMs tools [39,41].

#### Patient-Provider Communication and Relationship

The dynamics of communication and the relationship between patients and providers are identified as both barriers and facilitators [41,45]. The lack of patient-provider interaction and inadequate feedback are significant barriers, while strong relationships, interactivity, and timely communication facilitate implementation [41].

#### Personalization and Integration Into Clinical Workflow

The necessity for ePROMs tools to be personalized and seamlessly integrated into clinical workflows is a common theme across barriers and facilitators. Studies showed the reluctance to adopt new practices due to personalization and integration issues [38,41], contrasting with facilitators that advocate for straightforward data presentation and interoperability [42].

#### Technical Challenges and Support

Studies identified technical difficulties, including data entry, information retrieval, and a lack of technical support, as significant barriers [39]. Facilitators highlighted in the studies were ongoing technical support and user-friendly interfaces [47].

#### Workload and Treatment Quality

Concerns related to the impact of ePROMs on workload and treatment quality are highlighted barriers [40,43]. The increased workload for providers and potential distractions from acute health issues pose major barriers, while the potential for improved treatment quality through enhanced patient contexts offers a significant advantage [45].

### Quantitative Results

#### Overview

We present an overview of all quantitative results in Table 4.

**Table 4:** Quantitative results.

Study	Intervention and outcome	Effect of the intervention	Results	Sample size
Steele Gray et al [43]	Adopting the ePRO^a^ tool on: Goal Tracker Health Status Scales and Outcome Measures	Null	No statistical differences were seen for pre- versus post means of overall or subscale scores of assessment of QoL^b^, patient assessment of chronic illness care, and patient activation measure in both the control and intervention groups, nor between control and intervention.	16
Staeheli et al [27]	Screening intervention on: Rates of unrecognized and undiagnosed mental health, depression, posttraumatic stress disorder, and risky drinking	Positive effect	This tablet-based electronic screening tool identified significantly higher rates of behavioral health disorders than those that have been previously reported for this patient population. Electronic risk screening using patient-reported outcome measures offers an efficient approach to improving the identification of behavioral health problems and improving the rates of follow-up care.	275
Ainsworth et al [15]	Digital interventions on: Feasibility, adherence, retention, asthma-specific QoL, and asthma control	Positive effect	Both the intervention group and the control group improved from baseline to 3 and 12-mo follow-up, with numerically larger improvements in the asthma-related patient-reported outcomes measuring QoL and symptom control (AQLQ^c^ and AQC^d^) at both time points. Patients in the intervention group who completed 3-mo follow-up measures had mean improvement in asthma-related QoL and in the control group, with the between-group score difference higher in the intervention group, indicating improvements in QoL. There was no difference in the number of patients who showed minimal clinically important difference improvement at 3 mo (AQLQ>0.5) across groups. The same was true at 12 mo. By 12 mo, the between-group difference had risen higher in the intervention group.	88
Steele Gray et al [45]	ePRO mobile app and portal system on: Enable goal-oriented care delivery and health-related QoL The secondary outcome: self-management	Null	Patients with ePRO combined with usual care demonstrated a nonsignificant decrease in QoL compared with usual care only. ePRO combined with usual care demonstrated a nonsignificant decrease in patient activation. No patterns were evident when exploring descriptive trends in outcomes related to ePRO user intensity. The qualitative data from this study suggest that patients wanted more of a coaching approach with more touch points and interactions to maintain momentum, particularly for patients who started strong but then fizzled out. This finding suggests the need to better calibrate coherence when implementing digital health solutions with diverse user groups over time.	44
Harle et al [28]	PRO^e^ data in an EHR^f^ on: Providers and patient satisfaction with chronic noncancer pain care	Null	During the PRO phase, patients’ mean overall ratings of their visits were not significantly different between groups Similarly, patients’ mean perceptions that their “doctor did everything he/she could to help you with your pain or discomfort” and confidence “that in the future your pain or discomfort will be well controlled” did not differ significantly between the control and intervention groups in either phase. The intervention group’s satisfaction increased slightly, but not significantly, by the end of the education phase as well as from the end of the education phase to the PRO phase completion. A similar result was observed for each of the 4 satisfaction subscale measures. Furthermore, after adjusting for baseline satisfaction, there were no differences observed between the intervention and control groups within phases. The largest control versus intervention difference was a 0.20 higher mean satisfaction with time spent during visits with patients with chronic pain in the intervention group at the end of the PRO phase. However, this difference only approached significance.	370
Kroenke et al [29]	PROMIS^g^ symptom scores to clinicians on: The PROMIS profile-29 includes scales (sleep, pain, anxiety, depression, and fatigue)	Null	There were no differences between feedback and control group patients. Simple feedback of symptom scores to primary care clinicians in the absence of additional systems support or incentives is not superior to usual care in improving symptom outcomes. Satisfaction did not differ between study groups.	256
Lear et al [30]	Internet-based self-management and symptom monitoring program on: Number of all-cause hospitalizations from the time of randomization to the end of 2 y. Secondary outcomes: hospital length of stay, QoL, self-management, and social support	Null	There were no differences between the 2 groups with respect to changes in QoL. Self-management significantly changed in favor of the internet CDM^h^ intervention in 4 of the 8 domains: skill and technique acquisition, self-monitoring and insight, social integration and support, and emotional well-being. Social support significantly changed in favor of the internet CDM intervention in 2 of the 5 domains: emotional and informational support and overall support index. An internet-based self-management program did not result in a significant reduction in hospitalization. However, fewer participants in the intervention group were admitted to the hospital or experienced the composite outcome of all-cause hospitalization or death. These findings suggest that the internet CDM program has the potential to augment primary care among patients with multiple chronic diseases.	229
Miranda et al [31]	ePRO tool on: Estimate the cost of ePRO tool with quality-adjusted life year. Resource use and effectiveness of the ePRO tool	Null	Compared with standard care, the ePRO intervention was associated with higher costs. The ePRO tool is not a cost-effective technology for routine assessment. Compared with standard care, the ePRO intervention was associated with fewer QALYs^i^ (−0.03). No statistical difference in health-related QoL between ePRO and usual care groups. The tool would be considered cost-effective if it yielded an improvement of at least 0.03 QALYs. Long-term and the societal impacts of ePRO were not included in this analysis. Further research is needed to better understand its impact on long-term outcomes and in real-world settings.	45
Baron and Duffecy [32]	Technology-assisted sleep extension on: Feasibility and preliminary efficacy of a technology-assisted sleep extension. Predictors of improvement in sleep and blood pressure	Positive effect	Technology-assisted sleep extension intervention is feasible and well liked in this population. there was significant improvement in sleep variables in the intervention compared to the self-management group for TST^j^. Intervention adherence, use, and participant feedback: participants in the intervention group wore the Fitbit for 85% to 100% of study days and completed 90% of coaching sessions. All participants reported that they liked the intervention, and ease of the intervention was reported as 4 and 5 by 8 out of 9 participants. One participant rated ease of the intervention as 3. Change in blood pressure: there were significantly greater changes for 24-h systolic blood pressure and diastolic blood pressure in the intervention group compared with the self-management group (*P*=.02).	16
Bauer et al [46]	Mobile health platform supporting collaborative care on: Feasibility and acceptability of a mobile health platform. Daily surveys of mood and medication and weekly 9-item Patient Health Questionnaire and Generalized Anxiety Disorder 7-item	Positive effect	The feasibility and acceptability of the mobile platform is supported by the high early response rate; however, attrition was steep.	17
Ramallo-Fariña et al [34]	Interventions of knowledge transfer and behavior modification on: Knowledge transfer and behavior modification	Positive effect	The PTI^k^ group is significantly more adherent to the diet recommendations, compared with UC^l^, after 12 mo of follow-up. No differences were found in medication adherence, compared with UC. Compared with UC, both PFI^m^ and CBI^n^ show statistically significant differences at 12 mo for depression and anxiety. These differences disappear at 24 mo because all groups of patients improved. The diabetes distress score improved significantly compared with the UC group for CBI at 12 mo and for PTI and PFI at 24 mo. Health-related QoL significantly improved for all intervention groups at 12 mo compared with UC; however, this difference was only sustained for PTI at 18 mo. Neuropathic symptom scores were significantly lower for the CBI group at 12 mo (*P*=.02) compared with the UC group (the analysis of nonimputed data led to a nonsignificant result. This difference disappeared at 24 mo). While average scores were higher than 9 (on a total of 10), in all dimensions, for the group educational sessions, satisfaction with the web platform and SMS text message obtained scores >8.	2334
Tamisier et al [35]	Telemonitoring intervention on: Treatment adherence, QoL, functional status in symptomatic patients, health outcomes short form, and the Pichot Fatigue Scale	Null	The mean nightly use of CPAP^o^ was similar in the 2 groups. Using adherence categories, the intention-to-treat analysis showed similar high CPAP adherence in the 2 groups. This was also found in the per-protocol analysis. Following 6 mo of CPAP treatment, both groups exhibited substantial improvements in daily fatigue and excessive sleepiness, with no significant differences between the groups. There was an improvement in QoL with CPAP treatment that was significant only in the 12-item short form survey mental component in the TM^p^ group but with no significant differences between groups. Lipid control, and more specifically total blood cholesterol levels, improved significantly more in the TM group. This improvement was mainly driven by a decrease in low-density lipoprotein cholesterol levels. However, there was no change in BMI. As expected, there were a much greater number of interventions in the TM group. In patients with obstructed sleep apnea and at least 1 intervention, there was an overall 59% increase in the number of physician interventions and 54% in the number of home care provider’s interventions.	206
Trick et al [36]	ACASI^q^ system on: Time burden for patients	Positive effect	An ACASI software system can be included in a patient visit and adds minimal time burden. The burden was greatest for older patients, interviews in Spanish, and for those with less computer exposure. A patient’s self-reported health had minimal impact on response times.	1670
Yanicelli et al [37]	Home telemonitoring system on: Self-care treatment adherence and rehospitalization	Positive effect	After the 3-mo follow-up period, significant differences were found in the intragroup analysis of the CG^r^, which highlights a decreased treatment adherence at the end of the study in this group. Regarding intergroup analysis, there were no significant differences in treatment adherence between groups. After the 3-mo follow-up period, the mean EHFScB^s^ indicates an improvement in self-care for patients from the IG^t^ and a decrease in self-care for patients from the CG. Similarly, the intragroup analysis pointed out to significant differences in self-care within both the groups. There was no significant difference in rehospitalizations between CG and IG.	30
Owen-Smith et al [33]	Automate PRO data collection on: Compare study-enhanced PRO completion rates to those in clinical care	Positive effect	Adherence to pain-related PRO data collection using our enhanced tiered approach was high. No demographic or clinical identifiers other than age were associated with differential response by modality. Using automated modalities is feasible and may facilitate better sustainability for regular PRO administration within health care systems.	632

^a^ePRO: electronic patient-reported outcome.

^b^QoL: quality of life.

^c^AQLQ: Asthma Quality of Life Questionnaire.

^d^AQC: acceptable quality level.

^e^PRO: patient-reported outcome.

^f^EHR: electronic health record.

^g^PROMIS: Patient-Reported Outcomes Measurement Information System.

^h^CDM: chronic disease management.

^i^QALY: quality-adjusted life year.

^j^TST: total sleep time.

^k^PTI: patient-therapist interaction.

^l^UC: usual care.

^m^PFI: Physical Function Index.

^n^CBI: cognitive behavioral intervention.

^o^CPAP: continuous positive airway pressure.

^p^TM: telemonitoring.

^q^ACASI: audio computer-assisted self-interview.

^r^CG: control group.

^s^EHFScB: European Heart Failure Self-care Behaviour scale.

^t^IG: intervention group.

#### Clinical Outcomes and Quality of Life

A tablet-based electronic screening tool significantly improved the identification of behavioral health problems, offering an efficient approach to behavioral health screening and follow-up health care [27]. Overall, 3 studies showed no improvement on symptoms [29,34,35,43], while another study indicated a positive association [32]. The use of ePROMs in asthma management showed improvements within group in asthma-related quality of life and symptom control but were not statistically significant between groups [15]. The adoption of an ePROMs tool did not demonstrate statistical differences in overall or subscale scores of quality of life, patient activation, or satisfaction with chronic pain health care across both control and intervention groups [29,43,45,47]. Quality of life was used as the dependent variable by 5 studies, 3 showed no improvements [30,35,45], 1 showed improvement [15], and 1 showed decline [31].

#### Self-Management and Health Behaviors

Some studies showed significant improvement in self-management [37], skill and technique acquisition, and social integration [30], as well as adherence to diet recommendations and mental health outcomes over time [34]. Two studies showed no improvements in patient activation [45] and self-management [33].

#### System-Level Outcomes

Two studies indicated that implementing ePROMs does not reduce rehospitalizations [30,37]. Moreover, compared to usual care, the implementation of ePROMs is associated with a drop in cost-effectiveness because the data collection targets all patients and not specific cases. In addition, differences in patients’ digital literacy may also explain this drop in cost-effectiveness [31]. The study by Staeheli et al [27] showed an improvement in follow-up care.

#### Feasibility and Acceptability

Four studies indicated that ePROMs’ implementation is feasible and achieves a good level of acceptability [15,32,33,46], while 2 studies showed no association with satisfaction [28,29]. One study focusing on mental health outcomes also highlighted significant feasibility and acceptability as well as high engagement [46]. However, it also reported a steep attrition in long-term data collection [46]. One study implementing an audio computer-assisted self-interview system showed minimal time burden and high feasibility for patients regardless of age, language, and computer literacy [36].

## Discussion

### Principal Findings

It is difficult to draw clear conclusions about the effects of ePROM implementation in PHC. However, combining insights from both qualitative and quantitative data on the implementation and effects of ePROMs yields a nuanced understanding of their potential and challenges. A lack of digital literacy and engagement seems to be a key barrier to effectiveness, and in quantitative studies, ePROMs were well received when researchers emphasized feasibility and acceptability and provided training and support. This suggests that improving digital literacy and ensuring user-friendly design and adequate support are crucial for enhancing patient engagement with ePROMs and their effectiveness. Qualitative findings highlighted that the lack of personalization and patient-provider communication were impactful. Quantitatively, interventions that allowed for personalized feedback, goal setting, and self-management support showed positive effects. This indicates that ePROMs that are personalized and facilitate or enhance communication between patients and providers can lead to better health outcomes and patient experiences.

Qualitative data provided insights into perceived benefits, such as increased confidence and motivation and improved person-centered health care, even when quantitative outcomes showed null effects regarding clinical metrics such as health status and quality of life. This suggests a complex relationship between perceived benefits and measurable health outcomes, indicating that ePROMs may impact aspects of health care and patient experience not fully captured by quantitative measures. It also underscores the nuanced effects of digital and behavioral interventions on self-management and behavioral outcomes, highlighting the importance of personalized, interactive approaches and the potential for digital platforms to support but not fully substitute for comprehensive health care strategies aimed at enhancing patient engagement and self-management capabilities [30,33,34,45]. Qualitative insights suggest that despite the enthusiasm for ePROMs and digital interventions, there are concerns about their long-term impact and cost-effectiveness, with quantitative data echoing these concerns. While qualitative data highlight benefits such as increased confidence and motivation and improved patient-centered care, these effects do not always translate into measurable clinical outcomes. This highlights the need for more comprehensive research into the long-term outcomes, cost-effectiveness, and broader societal utility of digital health interventions.

Our review found that most studies were conducted in the United States (9/22, 41%) and Canada (8/22, 36%). The relative emphasis on these regions means that the findings may be influenced by North American health care financing models and policy environments. For instance, in the United States, fragmented insurance coverage and variable reimbursement structures can influence the adoption of digital health tools. In Canada, publicly funded health care and provincial eHealth strategies might support ePROM implementation by offering centralized funding or infrastructure but can also slow adoption. Other high-income countries may have distinct funding mechanisms, regulatory requirements, or national digital health agendas that shape ePROM uptake differently. With most included studies conducted in North America, our results have limited generalizability. Health care systems worldwide vary in digital infrastructure, reimbursement models, and regulatory frameworks, influencing ePROMs adoption. Future research should explore cross-cultural differences and assess implementation in diverse settings to better inform global policy and practice.

The results of this study corroborate those of other systematic reviews. First, systematic reviews carried out in pediatric health care [13], oncology [48,49], or breast cancer treatment [14] also present divergent results about patient satisfaction, quality of health care, health outcomes, patient management, and patient health behavior. The authors of these studies conclude that differences in the context of health care and the quality of ePROM implementation make it difficult to generalize the results of the studies that have been identified. Ishaque et al [48] also associates this divergence to the low statistical power of most of the studies reviewed. Second, the authors also conclude that ePROM implementations have the potential to improve patient health, provided they consider the barriers and facilitators specific to each health care setting [14,48,50].

Some of our results also differ from those of systematic reviews that have looked at other health care contexts. Indeed, systematic reviews indicate a positive, clear, and well-supported relationship between the implementation of ePROMs and health benefits. For example, in pediatrics, the integration of ePROMs is associated with an increase in health-related quality of life and patient satisfaction [13], while in oncology, the detection of unidentified problems and the monitoring of treatment response are improved by the implementation of ePROMs [49]. This difference in results from our study can probably be explained by the greater heterogeneity of populations and treatments in PHC, pointing at the significant challenge to widespread ePROMs implementation and uptake in this setting.

### Limitations

Our study has several limitations. The heterogeneity of study designs and outcome measures precluded meta-analysis, necessitating a narrative synthesis. The quantitative studies included in our sample assessed a diverse range of health indexes using scales that were not based on a common metric, making it impossible to extract standardized results for quantification. Consequently, we interpreted their findings using the vote-counting method [51]. However, the vote-counting method has been criticized [51], particularly because it does not account for effect sizes, limiting comparability and resulting in lower precision than meta-analysis. Our qualitative data analysis was limited to a descriptive approach. To accurately capture the net effect of ePROM implementation, future studies should aim to minimize the influence of barriers while maximizing the impact of facilitators. To advance this understanding, we plan to conduct a meta-synthesis of the identified qualitative studies, offering a comprehensive analysis of barriers, facilitators, and their interrelationships.

In terms of public decision-making, we can question the real size of the social benefits of implementing ePROMs. Indeed, only 1 of the studies in our sample carried out a cost-benefit analysis [31], and none of them did so in an ecological manner. No study has compared the benefits of implementing ePROMs to those of another intervention with the same level of financial investment. Future research should conduct cost-benefit analyses in a real-world context to identify which sector would yield the greatest overall benefit from financial investment.

Success in implementing ePROMs in PHC appears to hinge on addressing digital literacy, ensuring personalization and meaningful patient-provider interactions, carefully integrating technology into clinical workflows, and conducting thorough research on their long-term impacts and cost-effectiveness. ePROM implementation research could conduct large‐scale, multisite randomized trials or pragmatic trials to compare ePROM implementation strategies; investigate long-term outcomes and sustainability (eg, cost‐utility analyses and ecological cost‐benefit comparisons); focus on diverse patient populations, especially underserved communities, to address health equity and digital literacy gaps; and use standardized outcome measures or validated ePROM instruments to facilitate cross‐study comparisons. By focusing on these areas, future efforts could better assess the benefits of digital health technologies for patients, providers, and health care systems.

## Appendix

Multimedia Appendix 1 PRISMA checklist.

Multimedia Appendix 2 Search strategy.

Multimedia Appendix 3 Digital tools used for electronic patient-reported outcome measures’ collection and implementation in electronic medical records.

## Abbreviations

ePROM: electronic patient-reported outcome measure
MMAT: Mixed Methods Appraisal Tool
PHC: primary health care
PICOS: Population, Intervention, Comparison, Outcomes, and Setting
PRISMA: Preferred Reporting Items for Systematic Reviews and Meta-Analyses
RE-AIM: reach, effectiveness, adoption, implementation, and maintenance
