# Supplementary material for: Electronic Implementation of Patient-Reported Outcome Measures in Primary Health Care: Mixed Methods Systematic Review
Source: J Med Internet Res. 2025 May 5;27:e63639. doi: 10.2196/63639 (PMC12089857; doi:10.2196/63639)
Supplement: Multimedia Appendix 2 [file jmir_v27i1e63639_app2.docx]

**Supplementary materials – Database search strategy**

**Medline (OVID)**

**Date of the search:** 15-08-2022

**Database limit:** no database limit has been applied

| **#** | **Search strategy** | **Results** |
| --- | --- | --- |
| 1 | ((digital or electronic or tele) adj1 (patient? OR self) adj2 (report* OR relate* OR declar* OR rating* OR assess* OR examin* OR monitor* OR administer* OR rate?) adj2 (outcome* OR symptom* OR treat* OR measur*)).ti,ab,kw,kf OR ePROM?.ti,ab,kw,kf OR "e-PROM?".ti,ab,kw,kf OR ePRO?.ti,ab,kw,kf OR "e-PRO?".ti,ab,kw,kf OR telePRO?.ti,ab,kw,kf OR telePROM?.ti,ab,kw,kf OR ((digital or electronic* or tele) adj2 (PROM* OR PRO?)).ti,ab,kw,kf | 1689 |
| 2 | Patient Reported Outcome Measures/ OR ((patient? OR self) adj2 (report* OR relate* OR declar* OR rating* OR assess* OR examin* OR monitor* OR administer* OR rate?) adj2 (outcome* OR symptom* OR treat* OR measur*)).ti,ab,kw,kf | 100566 |
| 3 | patient outcome assessment/ OR Outcome Assessment, Health Care/ | 86298 |
| 4 | Self Report/ OR Self Disclosure/ | 48346 |
| 5 | 3 AND 4 | 1064 |
| 6 | 2 OR 5 | 101036 |
| 7 | Computer Peripherals/ OR Software/ OR Mobile Applications/ OR User-Computer Interface/ OR Data Collection/ OR exp Computers, Handheld/ OR Telemedicine/ OR Electronics/ OR Electronic Health Records/ OR Internet/  OR Internet-Based Intervention/ OR Medical Records Systems, Computerized/ OR Hospital Information Systems/ OR Digital Technology/ | 400506 |
| 8 | mhealth.ti,ab,kw,kf OR "m health".ti,ab,kw,kf OR ehealth.ti,ab,kw,kf OR "e health".ti,ab,kw,kf OR Smartphone?.ti,ab,kw,kf OR (tablet adj1 (application? OR computer?)).ti,ab,kw,kf OR ((Cell OR Mobile OR smart) adj1 Phone?).ti,ab,kw,kf OR (mobile adj2 (app* OR technolog* OR device? OR health*)).ti,ab,kw,kf OR "touch-screen".ti,ab,kw,kf OR ((portable OR "hand-held") adj2 (device OR technology)).ti,ab,kw,kf OR ((app* OR web) adj1 based).ti,ab,kw,kf OR (digital adj2 technolog*).ti,ab,kw,kf OR (digital adj1 health).ti,ab,kw,kf OR Internet.ti,ab,kw,kf | 258454 |
| 9 | 7 OR 8 | 590013 |
| 10 | (6 AND 9) OR 1 | 7512 |
| 11 | Chronic Disease/ OR (Chronic* adj2 (Illness* OR Disease? OR condition?)).ti,ab,kw,kf OR exp Hypertension/ OR Hypertension?.ti,ab,kw,kf  OR high blood pressure?.ti,ab,kw,kf OR exp Depressive Disorder/ OR Depression/ OR Depress*.ti,ab,kw,kf OR Anxiety/ OR Anxiety Disorders/ OR Anxiety.ti,ab,kw,kf OR (Chronic* adj2 musculoskeletal).ti,ab,kw,kf OR Chronic Pain/ OR (Chronic adj2 Pain?).ti,ab,kw,kf OR Musculoskeletal Diseases/ OR Musculoskeletal Disease?.ti,ab,kw OR Arthritis, Rheumatoid/ OR Arthritis/ OR Arthritis.ti,ab,kw,kf OR Polyarthritis.ti,ab,kw,kf OR Osteoporosis/ OR Osteoporos*.ti,ab,kw,kf OR Pulmonary Disease, Chronic Obstructive/ OR COAD.ti,ab,kw,kf OR COPD.ti,ab,kw,kf OR ((obstructive OR obstruction?) adj2 (chronic*) adj2 (respiratory OR pulmonary OR lung OR airway OR airflow OR bronchopulmonary)).ti,ab,kw,kf OR Asthma/ OR "Asthma-Chronic Obstructive Pulmonary Disease Overlap Syndrome"/ OR Asthma?.ti,ab,kw,kf  OR Bronchitis, Chronic/ OR (Chronic adj2 Bronchitis).ti,ab,kw,kf OR exp Hepatitis, Chronic/ OR (Chronic adj2 Hepatitis).ti,ab,kw,kf OR Cardiovascular Diseases/ OR Cardiovascular Disease?.ti,ab,kw,kf OR Myocardial Infarction/ OR ((Myocardial OR heart) adj2 Infarct*).ti,ab,kw,kf OR Cardiovascular Stroke?.ti,ab,kw,kf OR Heart Attack?.ti,ab,kw,kf OR Atrial Fibrillation/ OR Atrial Fibrillation?.ti,ab,kw,kf OR exp Angina Pectoris/ OR Angina.ti,ab,kw,kf OR Heart Failure/ OR ((Heart OR Cardiac OR Myocardial) adj2 (failure OR insufficiency OR decompensation OR defects)).ti,ab,kw,kf OR exp stroke/  OR Stroke?.ti,ab,kw,kf OR Ischemic Attack, Transient/ OR (Transient adj2 Ischemi*).ti,ab,kw,kf OR Thyroid Diseases/ OR (Thyroid* adj2 (disorder? OR disease? OR deficiency OR abnormalit* OR anomal* OR dysfunction?)).ti,ab,kw,kf OR Gastroesophageal Reflux/ OR ((gastric OR gastroesophageal OR "gastro-esophageal" OR Stomach OR oesophageal) adj2 (regurgitation OR reflux)).ti,ab,kw,kf OR Heartburn/  OR heartburn.ti,ab,kw,kf OR Pyros*.ti,ab,kw,kf OR Stomach Ulcer/ OR ((Stomach OR Gastric) adj2 Ulcer*).ti,ab,kw,kf OR Irritable Bowel Syndrome/  OR ((Colon* OR bowel OR colitis) adj2 (irritable OR disease? OR spastic)).ti,ab,kw,kf OR Crohn Disease/ OR "Crohn* Disease".ti,ab,kw,kf  OR Colitis, Ulcerative/ OR ((chronic OR colitis OR colorectitis) adj1 (ulcerati* OR ulcerosa OR ulcerous)).ti,ab,kw,kf OR exp Colonic Diverticulosis/ OR Diverticulosis.ti,ab,kw,kf OR Kidney disease/ OR exp Renal Insufficiency/  OR ((Kidney OR renal) adj1 (disease? OR Insufficienc* OR failure?)).ti,ab,kw,kf  OR Diabetes Mellitus/ OR Diabetes Mellitus, Type 1/ OR Diabetes Mellitus, Type 2/ OR Diabete?.ti,ab,kw,kf OR Urinary Retention/ OR ((Chronic* OR retention) adj1 (urine OR urinary)).ti,ab,kw OR Lower Urinary Tract Symptoms/ OR Lower Urinary Tract.ti,ab,kw,kf OR Dementia/ OR Dementia?.ti,ab,kw,kf OR Alzheimer Disease/ OR Alzheimer*.ti,ab,kw,kf  OR Hyperlipidemias/ OR Hyperlipemia?.ti,ab,kw,kf OR Hypercholesterolemia/  OR Hypercholesterolemia?.ti,ab,kw,kf OR Hypercholesteremia?.ti,ab,kw,kf  OR ((High OR Elevated) adj1 Cholesterol).ti,ab,kw,kf OR (cancer? OR neoplasm? OR tumor).mp OR exp Obesity/ OR Obesit*.ti,ab,kw,kf OR Heart Valve Diseases/ OR valve problem?.ti,ab,kw,kf OR valve replacement.ti,ab,kw,kf | 8739732 |
| 12 | 10 AND 11 | 3403 |

**Embase (Embase.com)**

**Date of the search:** 15-08-2022

**Database limit:** results were limited to pre-publications and the Embase database only

| **#** | **Search strategy** | **Results** |
| --- | --- | --- |
| 1 | ((digital or electronic or tele) NEAR/1 (patient$ OR self) NEAR/2 (report* OR relate* OR declar* OR rating* OR assess* OR examin* OR monitor* OR administer* OR rate$) NEAR/2 (outcome* OR symptom* OR treat* OR measur*)):ti,ab,kw OR ePROM:ti,ab,kw OR ePROMS:ti,ab,kw OR "e-PROM":ti,ab,kw OR "e-PROMS":ti,ab,kw OR ePRO:ti,ab,kw OR ePROS:ti,ab,kw  OR "e-PRO":ti,ab,kw OR "e-PROS":ti,ab,kw OR telePRO:ti,ab,kw OR telePROS:ti,ab,kw OR telePROM:ti,ab,kw OR telePROMS:ti,ab,kw OR ((digital or electronic* or tele) NEAR/2 (PROM* OR PRO OR PROS)):ti,ab,kw | 2,664 |
| 2 | 'patient-reported outcome'/de OR ((patient$ OR self) NEAR/2 (report* OR relate* OR declar* OR rating* OR assess* OR examin* OR monitor* OR administer* OR rate$) NEAR/2 (outcome* OR symptom* OR treat* OR measur*)):ti,ab,kw | 146,562 |
| 3 | 'outcome assessment'/de | 715,929 |
| 4 | 'self report'/de OR 'self disclosure'/de | 147,177 |
| 5 | #3 AND #4 | 10,252 |
| 6 | #2 OR #5 | 154,022 |
| 7 | 'computer'/de OR 'computer interface'/de OR 'software'/de OR 'mobile application'/exp OR 'information processing'/de OR 'electronics'/de OR 'electronic health record'/de OR 'personal digital assistant'/de OR 'mobile phone'/exp OR 'web-based intervention'/de OR 'Internet'/de OR 'telemedicine'/de OR 'telehealth'/de OR 'electronic medical record system'/de OR 'medical information system'/de OR 'digital technology'/de | 883,969 |
| 8 | mhealth:ti,ab,kw OR "m health":ti,ab,kw OR ehealth:ti,ab,kw OR "e health":ti,ab,kw OR Smartphone$:ti,ab,kw OR (tablet NEAR/1 (application$ OR computer$)):ti,ab,kw OR ((Cell OR Mobile OR smart) NEAR/1 Phone$):ti,ab,kw OR (mobile NEAR/2 (app* OR technolog* OR device$ OR health*)):ti,ab,kw OR "touch-screen":ti,ab,kw OR ((portable OR "hand-held") NEAR/2 (device OR technology)):ti,ab,kw OR ((app* OR web) NEAR/1 based):ti,ab,kw OR (digital NEAR/2 technolog*):ti,ab,kw OR (digital NEAR/1 health):ti,ab,kw OR Internet:ti,ab,kw | 325,463 |
| 9 | #7 OR #8 | 1,076,094 |
| 10 | (#6 AND #9) OR #1 | 14,249 |
| 11 | 'chronic disease'/exp OR (Chronic* NEAR/2 (Illness* OR Disease$ OR condition$)):ti,ab,kw OR 'hypertension'/exp OR Hypertension$:ti,ab,kw OR "high blood pressure$":ti,ab,kw OR 'depression'/exp OR Depress*:ti,ab,kw OR 'anxiety'/de OR 'anxiety disorder'/de OR Anxiety:ti,ab,kw  OR (Chronic* NEAR/2 musculoskeletal):ti,ab,kw OR 'chronic pain'/de OR (Chronic NEAR/2 Pain$):ti,ab,kw OR 'musculoskeletal disease'/de OR "Musculoskeletal Diseases":ti,ab,kw OR 'rheumatoid arthritis'/de OR 'chronic arthritis'/de OR Arthritis:ti,ab,kw OR Polyarthritis:ti,ab,kw OR 'osteoporosis'/de OR Osteoporos*:ti,ab,kw OR 'chronic obstructive lung disease'/de OR COAD:ti,ab,kw OR COPD:ti,ab,kw OR ((obstructive OR obstruction$) NEAR/2 (chronic*) NEAR/2 (respiratory OR pulmonary OR lung OR airway OR airflow OR bronchopulmonary)):ti,ab,kw OR 'asthma'/de  OR 'severe persistent asthma'/de OR Asthma$:ti,ab,kw OR 'chronic bronchitis'/exp OR (Chronic NEAR/2 Bronchitis):ti,ab,kw OR 'chronic hepatitis'/exp OR 'chronic hepatitis'/exp OR (Chronic NEAR/2 Hepatitis):ti,ab,kw OR 'cardiovascular disease'/de OR "Cardiovascular Disease$":ti,ab,kw OR 'heart infarction'/de OR ((Myocardial OR heart) NEAR/2 Infarct*):ti,ab,kw OR "Cardiovascular Stroke$":ti,ab,kw OR Heart Attack$:ti,ab,kw OR 'atrial fibrillation'/exp OR ((Atrial OR atrium) NEAR/2 Fibrillation$):ti,ab,kw OR 'angina pectoris'/de OR Angina:ti,ab,kw  OR 'heart failure'/de OR ((Heart OR Cardiac OR Myocardial) NEAR/2 (failure OR insufficiency OR decompensation OR defects)):ti,ab,kw OR 'cerebrovascular accident'/exp OR Stroke$:ti,ab,kw OR 'transient ischemic attack'/de OR (Transient NEAR/2 Ischemi*):ti,ab,kw OR 'thyroid disease'/de  OR (Thyroid* NEAR/2 (disorder$ OR disease$ OR deficiency OR abnormalit* OR anomal* OR dysfunction$)):ti,ab,kw OR 'gastroesophageal reflux'/de  OR ((gastric OR gastroesophageal OR "gastro-esophageal" OR Stomach OR oesophageal) NEAR/2 (regurgitation OR reflux)):ti,ab,kw OR 'heartburn'/de  OR heartburn:ti,ab,kw OR Pyros*:ti,ab,kw OR 'stomach ulcer'/de OR ((Stomach OR Gastric) NEAR/2 Ulcer*):ti,ab,kw OR 'irritable colon'/de OR ((Colon* OR bowel OR colitis) NEAR/2 (irritable OR disease$ OR spastic)):ti,ab,kw OR 'Crohn disease'/exp OR "Crohn* Disease":ti,ab,kw  OR 'ulcerative colitis'/exp OR ((chronic OR colitis OR colorectitis) NEAR/1 (ulcerati* OR ulcerosa OR ulcerous)):ti,ab,kw OR 'colon diverticulosis'/de  OR Diverticulosis:ti,ab,kw OR 'kidney disease'/de OR 'chronic kidney failure'/exp  OR ((Kidney OR renal) NEAR/1 (disease$ OR Insufficienc* OR failure$)):ti,ab,kw OR 'diabetes mellitus'/de OR 'insulin dependent diabetes mellitus'/de OR 'non insulin dependent diabetes mellitus'/de OR Diabete$:ti,ab,kw OR 'urine retention'/de OR ((Chronic* OR retention) NEAR/1 (urine OR urinary)):ti,ab,kw OR 'lower urinary tract symptom'/de OR "Lower Urinary Tract":ti,ab,kw OR 'dementia'/de OR Dementia$:ti,ab,kw OR 'Alzheimer disease'/de OR Alzheimer*:ti,ab,kw OR 'hyperlipidemia'/de OR Hyperlipemia$:ti,ab,kw OR 'hypercholesterolemia'/de OR Hypercholesterolemia$:ti,ab,kw OR Hypercholesteremia$:ti,ab,kw OR ((High OR Elevated) NEAR/1 Cholesterol):ti,ab,kw OR (cancer$ OR neoplasm$ OR tumor):ti,ab,kw,de OR 'obesity'/exp OR Obesit*:ti,ab,kw OR 'valvular heart disease'/de OR "valve problem$":ti,ab,kw OR "valve replacement":ti,ab,kw | 9,645,337 |
| 12 | #10 AND #11 | 4,689 |
| 13 | #12 AND [embase]/lim NOT ([embase]/lim AND [medline]/lim) | 2,572 |

**CINAHL (EBSCO)**

**Date of the search:** 15-08-2022

**Database limit:** no database limit has been applied

| **#** | **Search strategy** | **Results** |
| --- | --- | --- |
| 1 | TI ((digital or electronic or tele) N1 (patient# OR self) N2 (report* OR relate* OR declar* OR rating* OR assess* OR examin* OR monitor* OR administer* OR rate#) N2 (outcome* OR symptom* OR treat* OR measur*))  OR AB ((digital or electronic or tele) N1 (patient# OR self) N2 (report* OR relate* OR declar* OR rating* OR assess* OR examin* OR monitor* OR administer* OR rate#) N2 (outcome* OR symptom* OR treat* OR measur*)) OR TI ePROM# OR AB ePROM# OR TI "e-PROM#" OR AB "e-PROM#"  OR TI ePRO# OR AB ePRO# OR TI "e-PRO?" OR AB "e-PRO?" OR TI telePRO# OR AB telePRO# OR TI telePROM# OR AB telePROM# OR TI ((digital or electronic* or tele) N2 (PROM* OR PRO#)) OR AB ((digital or electronic* or tele) N2 (PROM* OR PRO#)) | 1,048 |
| 2 | MH "Patient-Reported Outcomes" OR TI ((patient# OR self) N2 (report* OR relate* OR declar* OR rating* OR assess* OR examin* OR monitor* OR administer* OR rate#) N2 (outcome* OR symptom* OR treat* OR measur*)) OR AB ((patient# OR self) N2 (report* OR relate* OR declar* OR rating* OR assess* OR examin* OR monitor* OR administer* OR rate#) N2 (outcome* OR symptom* OR treat* OR measur*)) | 60,966 |
| 3 | MH "Outcome Assessment" OR MH "Patient Assessment" | 68,227 |
| 4 | MH "Self Report" OR MH "Self Assessment" OR MH "Self Disclosure" | 91,087 |
| 5 | S3 AND S4 | 2,070 |
| 6 | S2 OR S5 | 62,339 |
| 7 | MH "Computer Peripherals" OR MH "User-Computer Interface" OR MH Software OR MH "Mobile Applications" OR MH "Internet-Based Intervention"  OR MH Internet OR MH "Electronic Health Records" OR MH "Computers, Hand-Held+" OR MH "Data Collection" OR MH Telemedicine OR MH Telehealth OR MH Electronics OR MH "Digital Technology" OR MH "Data Collection, Computer Assisted" OR MH "Patient Record Systems" | 217,084 |
| 8 | TI mhealth OR AB mhealth OR TI "m health" OR AB "m health" OR TI ehealth OR AB ehealth OR TI "e health" OR AB "e health" OR TI Smartphone# OR AB Smartphone# OR TI (tablet N1 (application# OR computer#)) OR AB (tablet N1 (application# OR computer#))  OR TI ((Cell OR Mobile OR smart) N1 Phone#) OR AB ((Cell OR Mobile OR smart) N1 Phone#) OR TI (mobile N2 (app* OR technolog* OR device# OR health*)) OR AB (mobile N2 (app* OR technolog* OR device# OR health*))  OR TI "touch-screen" OR AB "touch-screen" OR TI ((portable OR "hand-held") N2 (device OR technology)) OR AB ((portable OR "hand-held") N2 (device OR technology)) OR TI ((app* OR web) N1 based) OR AB ((app* OR web) N1 based) OR TI (digital N2 technolog*) OR AB (digital N2 technolog*) OR TI (digital N1 health) OR AB (digital N1 health) OR TI Internet OR AB Internet | 108,312 |
| 9 | S7 OR S8 | 288,274 |
| 10 | (S6 AND S9) OR S1 | 5,257 |
| 11 | MH "Chronic Disease" OR TI (Chronic* N2 (Illness* OR Disease# OR condition#)) OR AB (Chronic* N2 (Illness* OR Disease# OR condition#)) OR MH "Hypertension+" OR TI Hypertension# OR AB Hypertension#  OR TI "high blood pressure#" OR AB "high blood pressure#" OR MH Depression OR TI Depress* OR AB Depress* OR MH Anxiety  OR MH "Anxiety Disorders" OR MH "Generalized Anxiety Disorder"  OR TI Anxiety OR AB Anxiety OR TI (Chronic* N2 musculoskeletal) OR AB (Chronic* N2 musculoskeletal) OR MH "Chronic Pain" OR TI (Chronic N2 Pain#) OR AB (Chronic N2 Pain#) OR MH "Musculoskeletal Diseases"  OR TI "Musculoskeletal Disease#" OR AB "Musculoskeletal Disease#"  OR MH "Arthritis, Rheumatoid" OR MH Arthritis OR TI Arthritis OR AB Arthritis OR TI Polyarthritis OR AB Polyarthritis OR MH Osteoporosis  OR TI Osteoporos* OR AB Osteoporos* OR MH "Pulmonary Disease, Chronic Obstructive+" OR TI COAD OR AB COAD OR TI COPD OR AB COPD  OR TI ((obstructive OR obstruction?) N2 (chronic*) N2 (respiratory OR pulmonary OR lung OR airway OR airflow OR bronchopulmonary))  OR AB ((obstructive OR obstruction?) N2 (chronic*) N2 (respiratory OR pulmonary OR lung OR airway OR airflow OR bronchopulmonary))  OR MH Asthma OR MH "Asthma-Chronic Obstructive Pulmonary Disease Overlap Syndrome" OR TI Asthma# OR AB Asthma# OR MH "Bronchitis, Chronic" OR TI (Chronic N2 Bronchitis) OR AB (Chronic N2 Bronchitis) OR MH "Hepatitis, Chronic+" OR TI (Chronic N2 Hepatitis) OR AB (Chronic N2 Hepatitis) OR MH "Cardiovascular Diseases"  OR TI "Cardiovascular Disease#" OR AB "Cardiovascular Disease#"  OR MH "Myocardial Infarction" OR TI ((Myocardial OR heart) N2 Infarct*) OR AB ((Myocardial OR heart) N2 Infarct*) OR TI "Cardiovascular Stroke#" OR AB "Cardiovascular Stroke#" OR TI "Heart Attack#" OR AB "Heart Attack#"  OR MH "Atrial Fibrillation" OR TI "Atrial Fibrillation#" OR AB "Atrial Fibrillation#" OR MH "Angina Pectoris+" OR TI Angina OR AB Angina  OR MH "Heart Failure" OR TI ((Heart OR Cardiac OR Myocardial) N2 (failure OR insufficiency OR decompensation OR defects)) OR AB ((Heart OR Cardiac OR Myocardial) N2 (failure OR insufficiency OR decompensation OR defects))  OR MH "Stroke+" OR MH "Ischemic Stroke+" OR TI Stroke# OR AB Stroke#  OR MH "Cerebral Ischemia, Transient" OR TI (Transient N2 Ischemi*) OR AB (Transient N2 Ischemi*) OR MH "Thyroid Diseases" OR (Thyroid* N2 (disorder# OR disease# OR deficiency OR abnormalit* OR anomal* OR dysfunction#)) OR MH "Gastroesophageal Reflux" OR TI ((gastric OR gastroesophageal OR "gastro-esophageal" OR Stomach OR oesophageal) N2 (regurgitation OR reflux)) OR AB ((gastric OR gastroesophageal OR "gastro-esophageal" OR Stomach OR oesophageal) N2 (regurgitation OR reflux)) OR MH Heartburn OR TI heartburn OR AB heartburn  OR TI Pyros* OR AB Pyros* OR TI ((Stomach OR Gastric) N2 Ulcer*) OR AB ((Stomach OR Gastric) N2 Ulcer*) OR MH "Irritable Bowel Syndrome"  OR TI ((Colon* OR bowel OR colitis) N2 (irritable OR disease? OR spastic))  OR AB ((Colon* OR bowel OR colitis) N2 (irritable OR disease? OR spastic))  OR MH "Crohn Disease" OR TI "Crohn* Disease" OR AB "Crohn* Disease"  OR MH "Colitis, Ulcerative" OR TI ((chronic OR colitis OR colorectitis) N1 (ulcerati* OR ulcerosa OR ulcerous)) OR AB ((chronic OR colitis OR colorectitis) N1 (ulcerati* OR ulcerosa OR ulcerous)) OR MH "Diverticulum, Colon" OR TI Diverticulosis OR AB Diverticulosis OR MH "Kidney Diseases"  OR MH "Renal Insufficiency, Chronic" OR MH "Kidney Failure, Chronic"  OR TI ((Kidney OR renal) N1 (disease# OR Insufficienc* OR failure#))  OR AB ((Kidney OR renal) N1 (disease# OR Insufficienc* OR failure#)) OR MH "Diabetes Mellitus" OR MH "Diabetes Mellitus, Type 1" OR MH "Diabetes Mellitus, Type 2" OR TI Diabete# OR AB Diabete# OR MH "Urinary Retention" OR TI ((Chronic* OR retention) N1 (urine OR urinary)) OR AB ((Chronic* OR retention) N1 (urine OR urinary)) OR MH "Urinary Tract"  OR TI "Lower Urinary Tract" OR AB "Lower Urinary Tract" OR MH Dementia  OR TI Dementia# OR AB Dementia# OR MH "Alzheimer's Disease"  OR TI Alzheimer* OR AB Alzheimer* OR MH Hyperlipidemia OR TI Hyperlipemia# OR AB Hyperlipemia# OR TI Hyperlipidemia# OR TI Hyperlipidemia# OR MH Hypercholesterolemia OR TI Hypercholesterolemia# OR AB Hypercholesterolemia# OR TI ((High OR Elevated) N1 Cholesterol) OR AB ((High OR Elevated) N1 Cholesterol) OR TX (cancer# OR neoplasm# OR tumor) OR MH "Obesity+" OR TI Obesit* OR AB Obesit*  OR MH "Heart Valve Diseases" OR TI "valve problem#" OR AB "valve problem#" OR TI "valve replacement" OR AB "valve replacement" | 2,333,065 |
| 12 | S10 AND S11 | 2,474 |

**Web of Science**

**Date of the search:** 15-08-2022

**Database limit:** no database limit has been applied

| **#** | **Search strategy** | **Results** |
| --- | --- | --- |
| 1 | TS=((digital or electronic or tele) NEAR/1 (patient$ OR self) NEAR/2 (report* OR relate* OR declar* OR rating* OR assess* OR examin* OR monitor* OR administer* OR rate$) NEAR/2 (outcome* OR symptom* OR treat* OR measur*)) OR TS=(ePROM$) OR TS=("e-PROM$") OR TS=(ePRO$) OR TS=("e-PRO$") OR TS=(telePRO$) OR TS=(telePROM$) OR TS=((digital or electronic* or tele) NEAR/2 (PROM* OR PRO$)) | 8,351 |
| 2 | TS=((patient$ OR self) NEAR/2 (report* OR relate* OR declar* OR rating* OR assess* OR examin* OR monitor* OR administer* OR rate$) NEAR/2 (outcome* OR symptom* OR treat* OR measur*)) | 151,827 |
| 3 | TS=(mhealth) OR TS=("m health") OR TS=(ehealth) OR TS=("e health") OR TS=(Smartphone$) OR TS=(tablet NEAR/1 (application$ OR computer$)) OR TS=((Cell OR Mobile OR smart) NEAR/1 Phone$) OR TS=(mobile NEAR/2 (app* OR technolog* OR device$ OR health*)) OR TS="touch-screen" OR TS=((portable OR "hand-held") NEAR/2 (device OR technology)) OR TS=((app* OR web) NEAR/1 based) OR TS=(digital NEAR/2 technolog*) OR TS=(digital NEAR/1 health) OR TS=(Internet) | 1,200,014 |
| 4 | (#2 AND #3) OR #1 | 14,099 |
| 5 | TS=(Chronic* NEAR/2 (Illness* OR Disease$ OR condition$)) OR TS=(Hypertension$) OR TS=("high blood pressure$")  OR TS=(Depress*) OR TS=(Anxiety) OR TS=(Chronic* NEAR/2 musculoskeletal) OR TS=(Chronic NEAR/2 Pain$) OR TS=("Musculoskeletal Diseases") OR TS=(Arthritis) OR TS=(Polyarthritis) OR TS=(Osteoporos*)  OR TS=(COAD) OR TS=(COPD) OR TS=((obstructive OR obstruction$) NEAR/2 (chronic*) NEAR/2 (respiratory OR pulmonary OR lung OR airway OR airflow OR bronchopulmonary)) OR TS=(Asthma$) OR TS=(Chronic NEAR/2 Bronchitis) OR TS=(Chronic NEAR/2 Hepatitis) OR TS=("Cardiovascular Disease$") OR TS=((Myocardial OR heart) NEAR/2 Infarct*) OR TS=("Cardiovascular Stroke$") OR TS=("Heart Attack$") OR TS=((Atrial OR atrium) NEAR/2 Fibrillation$) OR TS=(Angina)  OR TS=((Heart OR Cardiac OR Myocardial) NEAR/2 (failure OR insufficiency OR decompensation OR defects)) OR TS=(Stroke$) OR TS=(Transient NEAR/2 Ischemi*) OR TS=(Thyroid* NEAR/2 (disorder$ OR disease$ OR deficiency OR abnormalit* OR anomal* OR dysfunction$))  OR TS=((gastric OR gastroesophageal OR "gastro-esophageal" OR Stomach OR oesophageal) NEAR/2 (regurgitation OR reflux)) OR TS=(heartburn) OR TS=(Pyros*) OR TS=((Stomach OR Gastric) NEAR/2 Ulcer*) OR TS=((Colon* OR bowel OR colitis) NEAR/2 (irritable OR disease$ OR spastic)) OR TS=("Crohn* Disease") OR TS=((chronic OR colitis OR colorectitis) NEAR/1 (ulcerati* OR ulcerosa OR ulcerous)) OR TS=(Diverticulosis) OR TS=((Kidney OR renal) NEAR/1 (disease$ OR Insufficienc* OR failure$)) OR TS=(Diabete$)  OR TS=((Chronic* OR retention) NEAR/1 (urine OR urinary)) OR TS=("Lower Urinary Tract") OR TS=(Dementia$) OR TS=(Alzheimer*) OR TS=(Hyperlipemia$) OR TS=(Hypercholesterolemia$) OR TS=(Hypercholesteremia$) OR TS=((High OR Elevated) NEAR/1 Cholesterol)  OR TS=(cancer$ OR neoplasm$ OR tumor) OR TS=(Obesit*) OR TS=("valve problem$") OR TS=("valve replacement") | 8,877,036 |
| 6 | #4 AND #5 | 4,075 |
