# Supplementary material for: Electronic Implementation of Patient-Reported Outcome Measures in Primary Health Care: Mixed Methods Systematic Review
Source: J Med Internet Res. 2025 May 5;27:e63639. doi: 10.2196/63639 (PMC12089857; doi:10.2196/63639)
Supplement: Multimedia Appendix 3 [file jmir_v27i1e63639_app3.docx]

|  | Digital tool used for ePROM collection | | | | | | | | |  |
| --- | --- | --- | --- | --- | --- | --- | --- | --- | --- | --- |
| Study ID | 1. Internet or Website | 2. Computer (software) | 3. Mobile app | 4. Electronic messaging (email, SMS) | 5. Electronic health record | 6. Telehealth (telemedicine) | 7. Social media | 8. Connected devices | 9. Other system | Is the digital tool linked to a platform (EMRs, Web-based portal system…) |
| Yanicelli 2021 |  |  | X |  |  |  |  |  |  | YES |
| SteeleGray 2019 |  |  | X |  |  |  |  |  |  | YES |
| Tamisier 2020 |  |  |  |  |  |  | X |  |  | YES |
| Trick 2015 |  | X |  |  |  |  |  |  |  | YES |
| Ahmed 2021 |  |  |  | X |  |  |  |  |  | YES |
| Owen-Smith 2018 | X |  |  | X | X |  |  |  | X | YES |
| Harle 2016 |  |  | X |  | X |  |  |  |  | YES |
| Gray 2021 |  |  | X |  |  |  |  |  |  | NO |
| BezerraGiordan 2022 |  |  | X |  |  |  |  |  |  | YES |
| Gray 2016 |  |  | X |  |  |  |  |  |  | YES |
| Bauer 2018 |  |  | X |  |  |  |  |  |  | YES |
| Baron 2019 |  |  | X |  |  |  |  |  |  | NO |
| Ainsworth 2019 |  |  |  |  |  |  |  | X | X | YES |
| Hans 2018 |  |  | X |  |  |  |  |  |  | YES |
| Schoenthaler 2020 |  |  | X | X |  |  |  |  |  | NO |
| Ramallo-Farina 2021 | X |  |  |  |  |  |  |  |  | NO |
| Miranda 2022 |  |  | X |  |  |  |  |  |  | NO |
| Lear 2021 |  |  |  | X |  |  |  |  |  | NO |
| Kroenke 2018 |  |  |  |  | X |  |  |  |  | NO |
| IrfanKhan 2018 |  |  |  |  |  |  |  | X |  | YES |
| Staeheli 2017 |  |  | X |  |  |  |  |  |  | NO |
| Harle 2016 |  | X | X |  | X |  |  |  |  | YES |
